# Supplementary material for: Anthocyanins and Type 2 Diabetes: An Update of Human Study and Clinical Trial
Source: Nutrients. 2024 May 29;16(11):1674. doi: 10.3390/nu16111674 (PMC11174612; doi:10.3390/nu16111674)
Supplement: Supplementary file 1 [file nutrients-16-01674-s001.zip › nutrients-3012946-supplementary.pdf]

nutrients

MDPI

Supplementary Table S1. The summary of (A) clinical trials and (B) meta-analyses related to anthocyanins and diabetes.

A. CLINICAL TRIALS									
**Study (year)**	**Study Design and Comparator (if applicable)**	**Number of participants**	**Drug/Substance**	**Dosage [mg/d]**	**Condition**	**Treatment/ use duration**	**Endpoints/Measures**	**Outcomes**	**Limitations**
Christiansen (2023) [1]	a randomized controlled trial	59	fermented (FE) or non-fermented aronia extract (AE) provided as snack bars	893 mg or 533 mg anthocyanins	patients with T2DM	8 weeks	• cardiovascular risk factors	• no effects on blood pressure (BP), adiponectin, and high-sensitive C-reactive protein (hs-CRP)<br>• increased in HDL-cholesterol in the FAE group<br>• increase in triglyceride in the AE group	possible interference of raisins and coconut oil
Christiansen (2023) [2]	a randomized controlled trial	59	fermented (FE) or non-fermented (AE) aronia extract provided as snack bars	893 mg or 533 mg anthocyanins	patients with T2DM	8 weeks	• glycated hemoglobin (HbA1c), fructosamine, insulin, glucose, glucagon-like peptide-1, glucose-dependent insulinotropic peptide (GIP) and glucagon,<br>• oral glucose tolerance tests<br>• anthropometric measurements	• increased GIP levels in FAE group<br>• no effects on HbA1c, fructosamine, insulin, glucose, glucagon-like peptide-1, and glucagon, oral glucose tolerance tests, anthropometric measurements	participants were too well controlled regarding risk markers of diabetes
Yang (2021) [3]	a randomized controlled trial	160	purified anthocyanins	320 mg/daily	participants with prediabetes or newly diagnosed diabetes (40–75 years old)	12 weeks	• adipsin and visfatin levels<br>• markers of diabetes	• increase in serum adipsin (0.15 μg/mL)<br>• decrease in visfatin (−3.5 ng/mL)<br>• decrease in HbA1c 0.11%<br>• improvement of apolipoprotein A-1 (apoA-1) and apolipoprotein B (apo B)	no measures to evaluate dietary or physical activity changes
Tasic (2021) [4]	a prospective open-label clinical case-series study	143	Alixir 400 PROTECT® (Standardized Aronia L. Melanocarpa Extract Extract-SAE)	120 mg of anthocyanins/daily	patients with MetS and patients with MetS and confirmed T2DM	4 weeks	• clinical and biochemical parameters of diabetes	• reduction in TG<br>• positive effect on body weight, total cholesterol, low and high-density lipoproteins, blood pressure and glycemia	short-term follow-up

Njike (2021) [5]	a randomized controlled crossover trial	20	Oliv oil or smoothie consisting of ½ cup frozen blueberries and 1 cup (8 oz) low-fat vanilla yogurt and oliv oil (EVOO)	n/a	patietnts at risk T2DM	data evaluated before and 2 h after ingestion of smoothie	• effects on endothelial function (EF)	• EVOO improved EF<br>• No effects on BP	small sample size, possible interference of many substances
Nikbakht (2021) [6]	An open-label clinical trial	40	Medox®	320 mg of anthocyanins	patients with T2DM (n=12), T2DM-at-risk (n=14) and healthy individuals (n=14)	4 weeks	• Dietary inflammatory index (DII)<br>• Biomarkers of inflammation<br>• Lipid profile	• reduction in interleukin-6 (IL-6), interleukin-18 (IL-18), and tumor necrosis factor-α (TNF-α) in the T2DM group<br>• reduction in fasting blood glucose level (FBG) in the T2DM group<br>• reduction in uric acid in the T2DM-at-risk group	small sample size<br>no placebo group<br>short duration
Desai (2021) [7]	a randomized controlled trial	12	Montmorency tart cherry juice (MTCJ)	30 ml/day	patients with MetS	7 days	• cardio-metabolic biomarkers	• reduction in BP, FBG<br>• reduction in TC, LDL-c	small sample size, short duration
Chan (2021) [8]	a randomized, double-blind, placebo-controlled cross-over study	20	bilberry extracts	1.4g/day	T2DM patients	4 weeks	• biomarkers of glycemic control, lipid profile, antioxidant, and inflammatory status	• no effects on cardiovascular risk factors or antioxidant status<br>• tendency for improved glycemic control	to a short period to monitor changes in HbA1c
Zhang (2020) [9]	a randomized crossover clinical trial	35	Red Raspberry *(Rubus idaeus L.)*with or without Fructo-Oligosaccharide (FOS)	236.8mg of anthocyanins/day	adults with prediabetes and insulin resistance	4 weeks	• microbial metabolites	• increase urolithins, phenyl-γ-valerolactones, and phenolic acids<br>• increase microbial metabolites	possible interference of many substances
Stote (2019) [10]	a randomized, placebo-controlled crossover trial	17	blueberries with a higher-carbohydrate breakfast meal	140g of blueberries	healthy adults	one time intervention	• glucose metabolism, gastrointestinal hormone response, and perceived appetite	• no effects on glucose metabolism, glucagon-like peptide-1 (GLP-1), GIP, peptide YY (PYY)<br>• increase pancreatic polypeptide (PP)	small sample size
Solverson (2019) [11]	a randomized cross-over trial	36	whole mixed berries or mixed berry juice	77.3-218 mg of anthocyanins per day	overweight and obese adults with no history of T2DM	4 treatment periods, 8 days each	• insulin sensitivity	• lower insulin serum	combination of pre-feeding berries before the glucose tolerance test and with the glucose tolerance test
Milutinović (2019)	a randomized controlled trial	35	chokeberry juice	150 ml/day	T2DM patients	3 months	• lipid and glucose metabolism	• decrease LDL-c, serum creatinine, and HbA1c	lack of a parallel control group

[12]									
Curtis (2019) [13]	a double-blind, randomized controlled trial	115	blueberries	75/150 g of blueberries	MetS patients	6 months	• insulin resistance and cardiometabolic function	• no effects on insulin resistance<br>• improvement of endothelial function<br>• augmentation of systemic arterial stiffness<br>• In statin nonusers (n = 71), increase HDL-c	an open recruitment policy
**B. META-ANALYSES**									
**Study (year)**	**Study Design and Comparator**	**Number of trials (subjects)**	**Drug/Substance**	**Dosage [mg/d]**	**Condition**	**Treatment/ use duration**	**Endpoints/Measures**	**Outcomes**	**Limitations**
Mao (2023) [14]	Meta-analysis of randomized controlled trials	13 trials (703 subjects with T2DM)	Pure anthocyanins or extract	a median dose of 320 mg/day	patients with T2DM	Duration of the interventions 4 to 24 weeks	• at least one of the glycemic parameters, insulin resistance markers, and lipid profile, including FBG, 2-h postprandial glucose, HbA1c, fasting insulin, HOMA-IR, TG, TC, HDL-c, and LDL-c	• reduction in HbA1c, FBG, 2-h postprandial glucose, TG, and LDL-c<br>• no effects of anthocyanins on fasting insulin, HOMA-IR, TC, HDL-c, systolic BP, and diastolic BP<br>• anthocyanins from fruit extracts or powder showed a significantly higher effect in lowering HbA1c	n/a
Neyestani (2023) [15]	Meta-analysis of randomized controlled trials	47 trials	ACN supplement/extract	n/a	healthy subjects, patients at risk CVD, including T2DM patients	3 weeks to 24 months	• cardiometabolic risk factors	• reduction in HbA1c, FBG in T2DM patients;<br>• reduction in TG, TC, LDL-c<br>• increase HDL-c<br>• decrease BMI and % of body fat	mostly evaluated the short-term effects of ACN intake
Delpino (2022) [16]	Meta-analysis of randomized controlled trials	22 trials	blueberry and cranberry	n/a	Patient with T2DM, MetS, and healthy individuals	n/a	• type 2 diabetes parameters	• reduction in the fasting blood glucose by 17.72 mg/dl<br>• reduction in glycated hemoglobin by 0.32%<br>• no effects of anthocyanins on fasting insulin<br>• no beneficial effects on healthy individuals	only seven studies were conducted with individuals with diabetes alone<br><br>combined effects of many substances
Raimund (2020) [17]	Meta-analysis of randomized controlled trials	27 trials	pure polyphenol or polyphenol-rich mixtures	one study used pure anthocyanins (392 mg/day)	patients with moderate-to-high T2DM risk,	Duration of the interventions 4 weeks to 1 year	• glucose, HbA1c, insulin, pro-insulin, HOMA-IR, islet amyloid polypeptide	• reduction in the fasting blood glucose by 3.32 mg/dl<br>• reduction in glycated hemoglobin by 0.24%	combined effects of many substances

					patients with T2DM			(IAPP)/amylin, pro-IAPP/pro-amylin, glucagon, C-peptide	• no change in insulin and HOMA-IR	
Fallah (2020) [18]	Meta-analysis of randomized controlled trials	37 trials (2068 subjects)	Anthocyanins supplements, and anthocyanins derived from fruit	The dose of anthocyanins ranges from 6.50 to 1024 mg/day	MetS, obese, prediabetes, diabetes, and healthy individuals	Duration of the interventions 2 weeks to 6 months	• effects of dietary anthocyanins on biomarkers of glycemic control and glucose metabolism	• higher doses of anthocyanins (>300 mg/day) for more than 8 weeks significantly decreased levels of FBG, 2-h postprandial glucose (PPG), HbA1c, and HOMA-IR	different sources of dietary anthocyanins that vary in composition as well as the contents of other bioactive compounds

AE—aronia exctrat, apoA-1—apolipoprotein A-1, apoB—apolipoprotein B, BP—blood pressure, BMI—body mass index, CVD—cardiovascular diseases, EF—endothelial function, FBG—fasting blood glucose, FE—fermented, GIP—glucose-dependent insulinotropic peptide, GLP-1—glucagon-like peptide-1, HbA1c—hemoglobin A1c, HDL-c—high-density lipoprotein, HOMA-IR—homeostasis model assessment-insulin resistance, hs-CRP—high-sensitive C-reactive protein, IAPP—islet amyloid polypeptide, IL-6—interleukin 6, IL-18—interleukin 18, LDL-c—low-density lipoprotein, MetS—metabolic syndrome, n/a—not available or not applicable, PP—pancreatic polypeptide PPG—postprandial glucose, PYY—peptide YY, T2DM—type 2 diabetes mellitus, TC—total cholesterol, TG—triglycerides, TNF-α—tumor necrosis factor alpha

1. Christiansen, C.B.; Jeppesen, P.B.; Hermansen, K.; Gregersen, S. The Impact of an 8-Week Supplementation with Fermented and Non-Fermented Aronia Berry Pulp on Cardiovascular Risk Factors in Individuals with Type 2 Diabetes. *Nutrients* **2023**, *15*, doi:10.3390/nu15245094.
2. Christiansen, C.B.; Jeppesen, P.B.; Hermansen, K.; Gregersen, S. Aronia in the Type 2 Diabetes Treatment Regimen. *Nutrients* **2023**, *15*, doi:10.3390/nu15194188.
3. Yang, L.; Qiu, Y.; Ling, W.; Liu, Z.; Yang, L.; Wang, C.; Peng, X.; Wang, L.; Chen, J. Anthocyanins regulate serum adipsin and visfatin in patients with prediabetes or newly diagnosed diabetes: a randomized controlled trial. *Eur J Nutr* **2021**, *60*, 1935-1944, doi:10.1007/s00394-020-02379-x.
4. Tasic, N.; Jakovljevic, V.L.J.; Mitrovic, M.; Djindjic, B.; Tasic, D.; Dragisic, D.; Citakovic, Z.; Kovacevic, Z.; Radoman, K.; Zivkovic, V., et al. Black chokeberry Aronia melanocarpa extract reduces blood pressure, glycemia and lipid profile in patients with metabolic syndrome: a prospective controlled trial. *Mol Cell Biochem* **2021**, *476*, 2663-2673, doi:10.1007/s11010-021-04106-4.
5. Njike, V.Y.; Ayettey, R.; Treu, J.A.; Doughty, K.N.; Katz, D.L. Post-prandial effects of high-polyphenolic extra virgin olive oil on endothelial function in adults at risk for type 2 diabetes: A randomized controlled crossover trial. *Int J Cardiol* **2021**, *330*, 171-176, doi:10.1016/j.ijcard.2021.01.062.
6. Nikbakht, E.; Singh, I.; Vider, J.; Williams, L.T.; Vugic, L.; Gaiz, A.; Kundur, A.R.; Colson, N. Potential of anthocyanin as an anti-inflammatory agent: a human clinical trial on type 2 diabetic, diabetic at-risk and healthy adults. *Inflamm Res* **2021**, *70*, 275-284, doi:10.1007/s00011-021-01438-1.
7. Desai, T.; Roberts, M.; Bottoms, L. Effects of short-term continuous Montmorency tart cherry juice supplementation in participants with metabolic syndrome. *Eur J Nutr* **2021**, *60*, 1587-1603, doi:10.1007/s00394-020-02355-5.

8. Chan, S.W.; Chu, T.T.W.; Choi, S.W.; Benzie, I.F.F.; Tomlinson, B. Impact of short-term bilberry supplementation on glycemic control, cardiovascular disease risk factors, and antioxidant status in Chinese patients with type 2 diabetes. *Phytother Res* **2021**, *35*, 3236-3245, doi:10.1002/ptr.7038.
9. Zhang, X.; Sandhu, A.; Edirisinghe, I.; Burton-Freeman, B.M. Plasma and Urinary (Poly)phenolic Profiles after 4-Week Red Raspberry (Rubus idaeus L.) Intake with or without Fructo-Oligosaccharide Supplementation. *Molecules* **2020**, *25*, doi:10.3390/molecules25204777.
10. Stote, K.; Corkum, A.; Sweeney, M.; Shakerley, N.; Kean, T.; Gottschall-Pass, K. Postprandial Effects of Blueberry (Vaccinium angustifolium) Consumption on Glucose Metabolism, Gastrointestinal Hormone Response, and Perceived Appetite in Healthy Adults: A Randomized, Placebo-Controlled Crossover Trial. *Nutrients* **2019**, *11*, doi:10.3390/nu11010202.
11. Solverson, P.M.; Henderson, T.R.; Debelo, H.; Ferruzzi, M.G.; Baer, D.J.; Novotny, J.A. An Anthocyanin-Rich Mixed-Berry Intervention May Improve Insulin Sensitivity in a Randomized Trial of Overweight and Obese Adults. *Nutrients* **2019**, *11*, doi:10.3390/nu11122876.
12. Milutinovic, M.; Velickovic Radovanovic, R.; Savikin, K.; Radenkovic, S.; Arvandi, M.; Pesic, M.; Kostic, M.; Miladinovic, B.; Brankovic, S.; Kitic, D. Chokeberry juice supplementation in type 2 diabetic patients - impact on health status. *J Appl Biomed* **2019**, *17*, 218-224, doi:10.32725/jab.2019.020.
13. Curtis, P.J.; van der Velpen, V.; Berends, L.; Jennings, A.; Feelisch, M.; Umpleby, A.M.; Evans, M.; Fernandez, B.O.; Meiss, M.S.; Minnion, M., et al. Blueberries improve biomarkers of cardiometabolic function in participants with metabolic syndrome-results from a 6-month, double-blind, randomized controlled trial. *Am J Clin Nutr* **2019**, *109*, 1535-1545, doi:10.1093/ajcn/nqy380.
14. Mao, T.; Akshit, F.N.U.; Mohan, M.S. Effects of anthocyanin supplementation in diet on glycemic and related cardiovascular biomarkers in patients with type 2 diabetes: a systematic review and meta-analysis of randomized controlled trials. *Front Nutr* **2023**, *10*, 1199815, doi:10.3389/fnut.2023.1199815.
15. Neyestani, T.R.; Yari, Z.; Rasekhi, H.; Nikooyeh, B. How effective are anthocyanins on healthy modification of cardiometabolic risk factors: a systematic review and meta-analysis. *Diabetol Metab Syndr* **2023**, *15*, 106, doi:10.1186/s13098-023-01075-0.
16. Delpino, F.M.; Figueiredo, L.M.; Gonçalves da Silva, T.; Flores, T.R. Effects of blueberry and cranberry on type 2 diabetes parameters in individuals with or without diabetes: A systematic review and meta-analysis of randomized clinical trials. *Nutr Metab Cardiovasc Dis* **2022**, *32*, 1093-1109, doi:10.1016/j.numecd.2022.02.004.
17. Raimundo, A.F.; Félix, F.; Andrade, R.; García-Conesa, M.T.; González-Sarrías, A.; Gilsa-Lopes, J.; do Ó, D.; Raimundo, A.; Ribeiro, R.; Rodriguez-Mateos, A., et al. Combined effect of interventions with pure or enriched mixtures of (poly)phenols and anti-diabetic medication in type 2 diabetes management: a meta-analysis of randomized controlled human trials. *Eur J Nutr* **2020**, *59*, 1329-1343, doi:10.1007/s00394-020-02189-1.
18. Fallah, A.A.; Sarmast, E.; Jafari, T. Effect of dietary anthocyanins on biomarkers of glycemic control and glucose metabolism: A systematic review and meta-analysis of randomized clinical trials. *Food Res Int* **2020**, *137*, 109379, doi:10.1016/j.foodres.2020.109379.
